# 胸腔镜下肺段切除术治疗非小细胞肺癌的研究现状与进展

**DOI:** 10.3779/j.issn.1009-3419.2018.04.12

**Published:** 2018-04-20

**Authors:** 晓峰 陈, 宇龙 谈

**Affiliations:** 复旦大学附属华山医院胸外科 Department of Thoracic Surgery, Huashan Hospital Affiliated to Fudan University

近年中国的癌症流行病学统计中，肺癌已经成为患病率和致死率最高的癌症。在全球范围约有1.41千万新发癌症病例，其中肺癌病例有180万，占癌症发病总数的13%，同时也是癌症中诊断率最高的病种。全球男性、发达国家女性癌症死亡率最高的也是肺癌。中国女性的肺癌发病率为20.4/10万，该数据已超过部分欧洲国家（女性吸烟率较高的）^[1-3]^。

随着我国治疗实力与人民生活水平的提高，更多的非小细胞肺癌（non-small cell lung cancer, NSCLC）在早期能被胸部螺旋CT发现并及时治疗。使得早期NSCLC患者的生存率甚至治愈率大大提高。同时国内胸外科医生对电视辅助胸腔镜手术（video-assisted thoracic surgery, VATS）掌握日益熟练，其围术期的康复优势也更为体现，能在不降低远期预后的基础上减少疼痛、减少住院日、肺功能恢复更快、并发症更少。但是何种情况肺段切除术能达到根治性的目的，目前还是存在争议。

根据2017年美国国家综合癌症网（National Comprehensive Cancer Network, NCCN）治疗NSCLC临床实践指南的最新内容，可保留非组织很少或因不能耐受肺叶切除，周围型结节≤2 cm并至少符合下列一种标准：组织学类型为单纯原位腺癌、CT显示结节≥50%表现为毛玻璃样、影像学随访证实肿瘤倍增时间较长（≥400天），为肺段切除的适应症。并且提出有丰富VATS经验的高流量医学中心里，VATS可提高患者术后近期疗效。目前最新的NSCLC TNM分期是由国际肺癌研究学会（International Association for the Study of Lung Cancer, IASLC）2015年对肺癌分期系统进行了更新，称之为第八版的NSCLC TNM分期。相比于前版本分期，第八版的数据在已建立的TNM分期基础上进行前瞻性的分析，所形成的预后更加有指导作用。同时新的分期也更新了肺段切除术的适应症^[4-14]^。

## 1 肺段切除术治疗NSCLC远期预后研究进展

外科手术仍是早期NSCLC首选的治疗方法。解剖性肺叶切除术被认为是对大多数NSCLC的标准手术治疗方式。Kirby等^[15]^在20世纪90年代初期报道VATS肺叶切除术之后，该技术在全世界范围内逐渐推广。由于其手术安全性较好、并发症较少、切口更加美观，VATS肺叶切除术得到越来越多胸外科医生的青睐。肺段切除术则是首先由日本Murakami等^[16]^在治疗NSCLC中使用的，肺段切除术相比于肺叶切除术能够保留更多的肺组织从而对患者的肺功能影响较少。而相对于楔形切除术，肺段切除术则在解剖关系上能切除与病变组织关系密切的淋巴管道及淋巴结、血管及支气管，预后安全性较好。纵观外科手术治疗NSCLC，其中Ⅰ期和Ⅱ期患者的5年生存率约为53%-58%和48%-55%。但预后存在明显的人种差异。研究发现对于pN0分期的患者，亚洲患者5年生存率最好高达79%，而欧洲患者预后最差，仅为54%，由于新的分期没有考虑人群特征及地域差异，不同地域患者生存率及预后判断可能存在一定偏差^[17]^。

近年来围绕Ⅰ期NSCLC患者行肺段切除术与肺叶切除术的比较研究资料愈发完善。Moon等^[18]^比较了2000年-2014年14, 549例肺叶切除术和809例肺段切除术NSCLC患者，其肿瘤大小均≤2 cm，其5年生存率未见明显差异。对于 > 2 cm肿块NSCLC的报道，Cao等^[19]^从16, 819例肺癌术后患者预后回顾性分析得出，2.1 cm-3.0 cm大小的NSCLC患者5年生存率，肺叶切除术患者较肺段切除术及楔形切除术更佳。对于不能耐受肺叶切除术的患者，肺段切除术与楔形切除术比较未见差异。Bedetti等^[20]^将1990年-2016年比较肺段切除术和肺叶切除术的相关研究资料进行荟萃分析，共纳入24, 542例NSCLC患者，得出两种手术方式对Ⅰ期NSCLC患者治疗预后无差异性影响。然而目前为止，与之相关的随机对照实验且发表的仅有Ginsberg等^[21]^的研究，该研究提示亚肺叶切除术有更高的局部复发风险（17.2% *vs* 6.4%）以及更糟糕的远期生存率。不过该研究由于“亚肺叶切除术”包涵了楔形切除，纳入研究的NSCLC为T1的患者，肿瘤直径有些达到3 cm，并不能明确肺段切除术的根治性效果及适应症。

有关近期以中国患者为研究人群纳入的研究，Xiao等^[22]^比较2003年-2013年NSCLC Ⅰ期并≤3 cm患者接受肺段切除术的1, 156例和接受肺叶切除术的17, 748例，发现适当的淋巴结切除或取样的肺段切除术可以很好的替代肺叶切除术。

综上所述，在Ⅰ期NSCLC尤其是≤2 cm的患者行肺段切除术的获益受学术界认可度较高，而 > 2 cm且 < 5 cm的NSCLC是否适合肺段切除术仍有争议，尤其需要随机对照试验来明确肺段切除术远期预后的安全性。

## 2 肺段切除术治疗NSCLC近期康复研究进展

肺段切除术在解剖范围上相对肺叶切除术保留了更多的肺组织，对肺功能的保存方面以及因肺功能差不能耐受手术患者的手术机会都有积极作用。Keenan等^[23]^回顾性分析54例行肺段切除术和147例行肺叶切除术的Ⅰ期NSCLC患者。对比其术前和术后1年的用力肺活量（FVC）、1 s用力呼气容积（FEV_1_）、最大通气量（MVV）和一氧化碳弥散量（DLCO），发现即使术前肺段切除组比肺叶切除组患者肺功能更差，平均FEV_1_分别为75.1%和55.3%，术后1年肺叶切除组FVC、FEV_1_、MVV、DLCO均显著下降，而在肺段切除组仅DLCO下降明显，表明肺段切除术能更好地保护术后肺功能。对于术前肺功能差以及因各种疾病不能耐受手术的患者，肺段切除术是一个很好的选择^[24]^。但由于肺段切除术较肺叶切除术相对操作难度大，部分术者担忧在较少工作量的中心开展肺段切除术的获益不大。对此，Yao等^[25]^分享了相关工作经验，在2015年-2016年开展VATS肺段切除术的40例NSCLC cT1aN0M0患者的预后对比同期47例NSCLC cT1aN0M0行VATS肺叶切除术患者，发现两组手术时间、术中出血量、术后胸管引流量、住院天数以及术后并发症率无差异，但肺段切除术患者术后肺功能表现更佳。若有更加熟练的基础上，肺段切除术的近期手术获益能更加凸显。

## 3 肺段切除术不能被楔形切除术替代

相比于肺段切除术，楔形切除术没有充分的清理肿瘤区域相关的淋巴管道及淋巴结、血管、支气管，存在局部复发及转移的风险，被认为在多数NSCLC情况下不能达到根治效果的手术方式。但历年来一直有学者质疑肺段切除术对比于楔形切除术并没有远期更多的获益。近年来随着肺段解剖学的应用掌握在胸外科界的提高，更多真正解剖性肺段切除术的进行，才使得肺段切除术与楔形切除术的预后有差异性的表现。Dziedzic等^[26]^回顾性研究2007年-2013年共6, 905例NSCLC Ⅰ期患者，其分别行肺叶切除术、肺段切除术、楔形切除术。发现楔形切除术组的3年、5年生存率显著低于其他组。而肺段切除术组与肺叶切除术组并无差异。但是在早期NSCLC，尤其是在cT1N0M0患者中，肺段切除术的安全性“优势”并不一定能体现出来。Altorki等^[27]^回顾性对比了2000年-2014年cT1N0M0 NSCLC患者，其中160例行楔形切除术，129例行解剖性肺段切除术。研究发现两组3年、5年生存率并无统计学差异，即使解剖性肺段切除术进行了更多的淋巴结清扫。研究提示在T1期NSCLC酌情减少淋巴结清扫范围，以降低手术并发症如乳糜胸、淋巴瘘的发生可能是总体获益且安全的。Hou等^[28]^研究纳入1, 181例不耐受肺叶切除术的高危NSCLC Ⅰ期行肺段切除术患者和2, 003例相似情况行楔形切除术患者。通过荟萃分析发现NSCLC Ⅰ期上述患者，肺段切除术组总体生存率较高，但是≤2 cm的NSCLC肿块直径情况下，楔形切除术和肺段切除术预后相似。综上所述，在cT1N0M0尤其是肿块≤2 cm的NSCLC患者，楔形切除术和肺段切除术的获益比较仍需要随机对照实验证据的明确。

近年来围绕NSCLC Ⅰ期的VATS肺段切除术的研究提示其手术安全性和可靠性在逐步上升。这与解剖性肺段切除术的开展以及临床医生对VATS技术掌握提升的因素密不可分。在未来仍需要大量临床资料，尤其是大型随机对照实验来证明肺段切除术在 > 2 cm的Ⅰ期NSCLC患者中的应用是具有安全且获益的。
